# DNA methylation variability in pediatric aplastic anaemia contributes to T‐cell differentiation

**DOI:** 10.1002/ctm2.70588

**Published:** 2026-01-08

**Authors:** Junchen Lai, Fangli Chen, Yan Miao, Manpin Zhang, Hua Zhu, Huanhuan Liang, Liting Yang, Yingwen Zhang, Dabin Tang, Chengjuan Luo, Changying Luo, Yanxin Li, Xiaodong Wang, Yu Liu, Jing Chen, Xia Qin, Xinan Wang

**Affiliations:** ^1^ Blood and Marrow Transplantation Center Shanghai Children's Medical Center School of Medicine, Shanghai Jiao Tong University Shanghai China; ^2^ Department of Hematology Tongren Hospital Shanghai Jiao Tong University School of Medicine Shanghai China; ^3^ Laboratory Department II Shanghai Children's Medical Center School of Medicine, Shanghai Jiao Tong University Shanghai China; ^4^ Cell Therapy Center Shanghai Children's Medical Center School of Medicine, Shanghai Jiao Tong University Shanghai China; ^5^ Pediatric Translational Medicine Institute Department of Hematology & Oncology Shanghai Children's Medical Center National Health Committee Key Laboratory of Pediatric Hematology & Oncology School of Medicine, Shanghai Jiao Tong University Shanghai China; ^6^ Hainan Branch of Shanghai Children's Medical Center School of Medicine Shanghai Jiao Tong University Hainan China; ^7^ Shanghai Children's Medical Center School of Medicine Shanghai Jiao Tong University Shanghai China

1

Dear Editor,

The key findings suggest an association between DNA methylation variability and aberrant balance of Th17/Treg cells and T‐cell differentiation in children with aplastic anaemia (AA), which may be related to activation of the JAK/STAT signalling pathway. The CAMK4 subtype in CD4+ naïve T cells provided potential evidence that supports the ‘locust’ theory in the progression of pediatric AA and possibly novel targets for immunotherapy in the future.

AA is considered as an immune‐mediated bone marrow failure syndrome and exhibits an inexplicable peak of age distribution in children.^1^, ^2^ The incidence in East Asia is two to three times that of Western countries, suggesting significant differences in genetic background.^3^ T‐cell differentiation, which plays a critical role in disease pathogenesis, is governed by both genetic and epigenetic programs.^4^ So far, the role of DNA methylation in pediatric AA and its crosstalk with aberrant T‐ cell differentiation remains unexplored.

We recruited 83 patients with acquired AA and 22 controls (<18 years) between January 2016 and October 2024. Methods were detailed in the Supporting Information. This study was approved by the institutional review board of Shanghai Children's Medical Center and conducted in accordance with the Declaration of Helsinki.

We performed flow cytometry in 76 pediatric AA patients and 20 healthy controls to detect the populations of T cells, B cells, dendritic cells (DCs), and natural killer (NK) cells and their subtypes (Figure 1, Table S1, Figure S1a). The average age was 8.3 years for both AA patients (range 2–17) and controls (range 5–12; Table S2). Among the patients, 23 (30%) had very severe AA (VSAA), 20 (26%) had severe AA (SAA), and the remaining 33 (43%) had non‐severe AA (NSAA).

**FIGURE 1:**
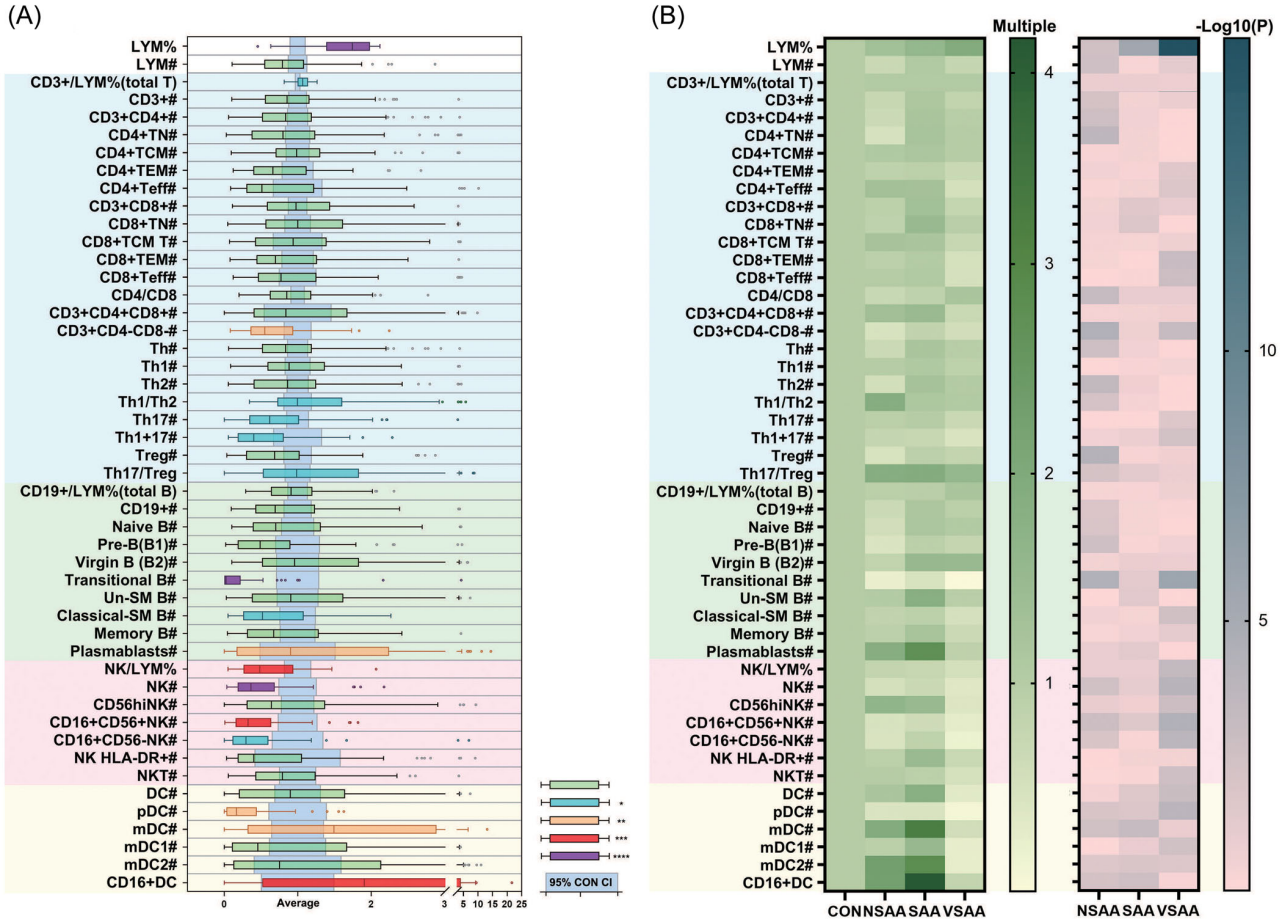
Immune cells in aplastic anaemia patients. (A) Percentages and absolute counts in patients. (B) Multiples of average and *p*‐value in NSAA, SAA, and VSAA cohorts, compared with the healthy control cohort. CI, confidence interval; classical‐SM B, classical‐switched memory B cells; CON, control; DC, dendritic cells; LYM, lymphocyte; mDC, myeloid dendritic cells; NK cells, natural killer cells; NSAA, non‐severe aplastic anaemia; pDC, plasmacytoid dendritic cells; SAA, severe aplastic anaemia; TCM, central memory T cells; TEM, effector memory T cells; Teff, effector T cells; TN, naïve T cells; Un‐SM B, un‐switched memory B cells; VSAA, very severe aplastic anaemia. *p < .05; **p < .01; ***p < .001; ****p < .0001.

The percentage of T cells in lymphocytes was 78.6% ± 7.8% versus 74.0% ± 5.4% in pediatric AA patients versus the controls (*p* = .015) and showed a trend of increase with disease severity (Figure 2A). The absolute T‐cell count did not differ in the two cohorts (Figure S1b). The percentage and absolute NK cell count were lower in AA patients than in controls (percentage: 7.1% ± 5.0% vs. 11.6% ± 4.8%, *p* = .0004; absolute count: 100 ± 87 × 10^6^/L vs. 198 ± 115 × 10^6^/L, *p* < .0001; Figure 2B,C). The percentage and absolute count in B cells and DCs did not differ between the two cohorts (Figure S1c,d and Figure 2D). The Th17/Treg ratio was 1.2 ± 1.2 versus.8 ± .3 in patients and controls, respectively (*p* = .022) and showed a trend of decrease with disease severity (Figure 2F and Figure S1e). There was no significant difference in CD4/CD8 ratio and percentages of naïve T cells (TN), central memory T cells (TCM), effector memory T cells (TEM), and effector T cells (Teff) subsets between patients and controls (Figure S1f,g).

**FIGURE 2:**
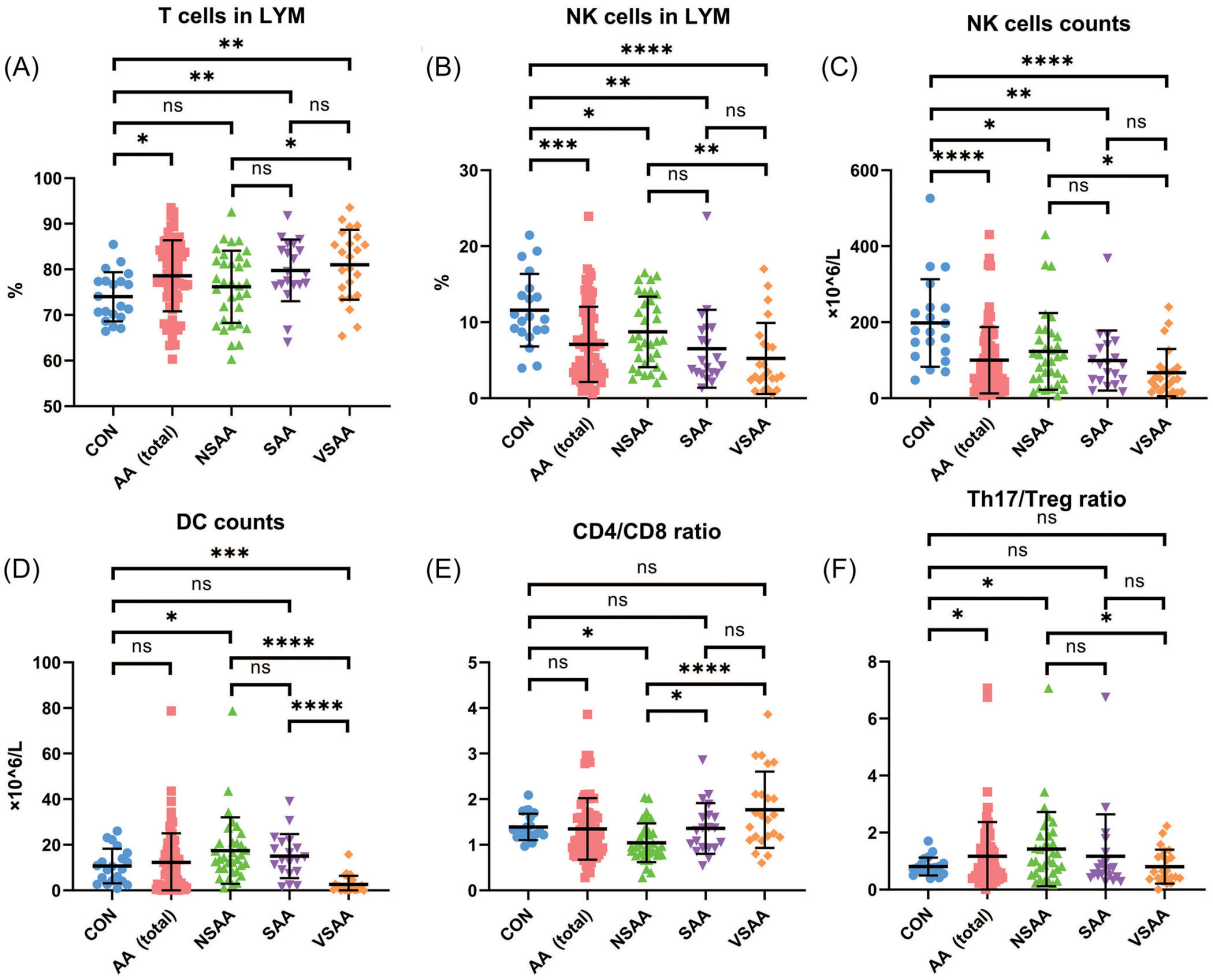
Immune cells in control and patient cohorts. (A) Percentages of T cells in lymphocytes. (B) NK cells in lymphocytes. (C) Absolute counts of NK cells. (D) Absolute counts of DC. (E) CD4+/CD8+ ratio. (F) Th17/Treg ratio. AA, aplastic anaemia; CON, control; DC, dendritic cells; LYM, lymphocyte; NK cells, natural killer cells; NSAA, non‐severe aplastic anaemia; SAA, severe aplastic anaemia; Th17, T helper cells 17; Treg, T regulatory cells; VSAA, very severe aplastic anaemia; WBC, whole blood cells. *p < .05; **p < .01; ***p < .001; ****p < .0001.

We performed whole‐genome bisulfite sequencing on CD3+ T cells from peripheral blood in five patients (three with VSAA and two with SAA) and two controls. The average DNA methylation did not differ between the two groups (Figure S2a–c). After removing probes located in introns and intergenic regions, over‐representation analysis (ORA) of the 2448 genes showed enrichment of differentially methylated regions (DMRs) in positive regulation of cell activation and small guanosine triphosphataseS (GTPases)‐mediated signal transduction by Gene Ontology (GO). Enriched pathways upon Kyoto Encyclopedia of Genes and Genomes analysis included the Th17 cell differentiation, Th1 and Th2 cell differentiation, and PD‐L1 expression and PD‐1 checkpoint pathways related to T‐cell differentiation (Figure 3A), with significant gene overlap among the three pathways (Figure 3B and Figure S2d,e). We also noticed hypomethylation of *STAT3* (Figure 3C,D).

**FIGURE 3:**
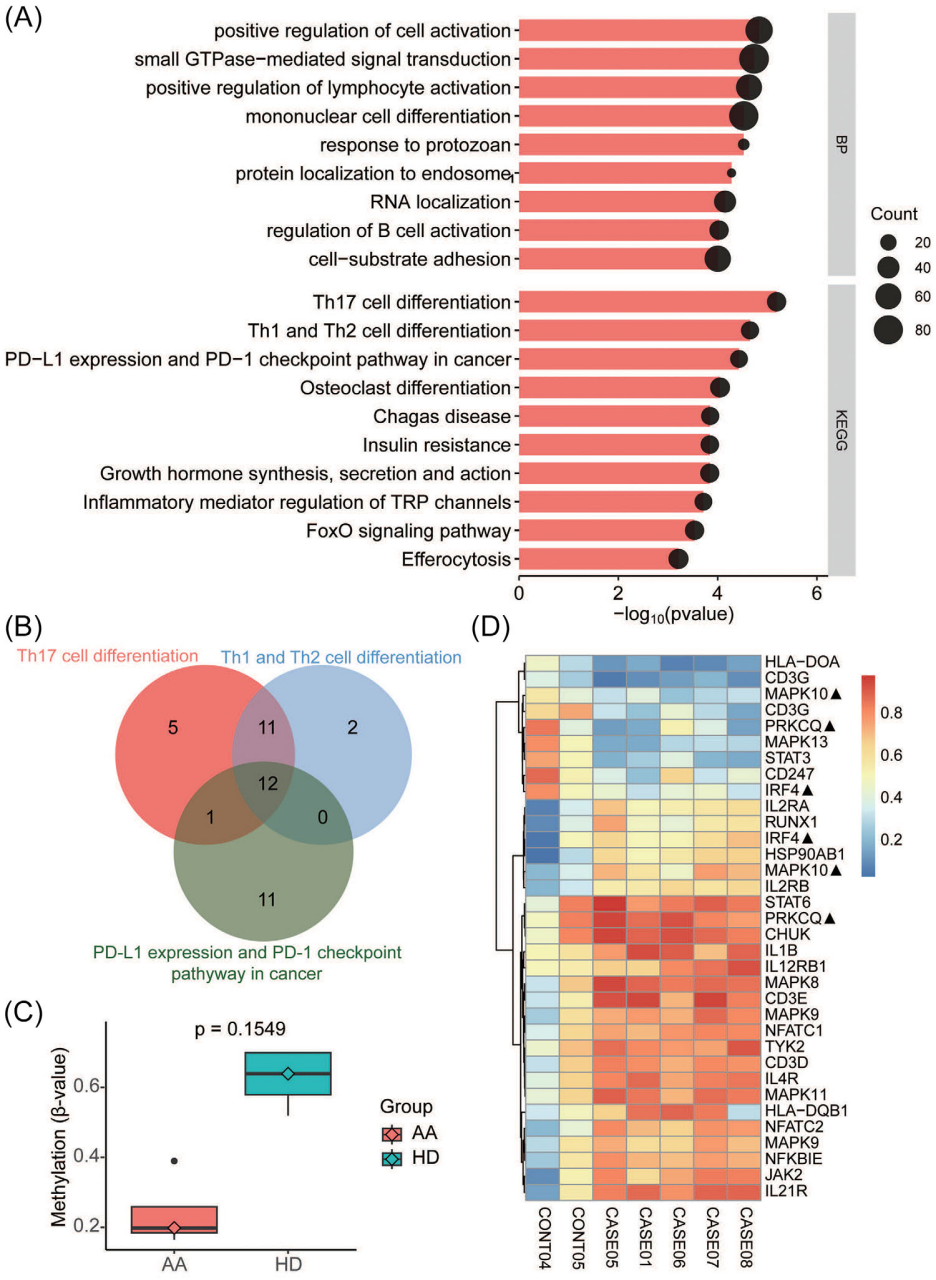
Differentially methylated regions (DMRs) enriched in th17 differentiation pathway. (A) Over‐representation analysis (ORA) of DMRs showed enrichment of T‐cell differentiation and cell activation. (B) Venn plot of genes from top 3 Kyoto Encyclopedia of Genes and Genomes (KEGG) enrichment pathways. (C) Boxplot of different methylation status of *STAT3* between aplastic anaemia (AA) patients and healthy controls. (D). Hierarchical heatmap of methylation of genes from the th17 differentiation pathway. ▲: Probes located in the same gene showed different methylation trends between AA patients and HD.

We performed single‐cell RNA sequencing (scRNA‐seq) from peripheral blood samples in two patients (both with VSAA) combined with the data of three healthy subjects from the Gene Expression Omnibus (GEO) database (GSM6250006). We annotated 55 939 cells into T cells (including CD4+T and CD8+T), B cells, monocytes, NK cells, monocytes and platelets (Figure 4A,B and Figure S3a). Then, we re‐clustered the CD4+ T cells, which increased in AA patients, into five main cell types: TN (CAMK4, MYC, SOX4, CD74, and CXCR4 subtypes), TCM, TEM, Treg, and Th17 (Figure 4C and Figure S3b,c). The proportion of C01‐CAMK4‐TN was higher in AA patients, while that of Treg cells was lower (Figure 4D). The ORA of the C01‐CAMK4‐TN subcluster shared the enrichment of PD‐L1 expression and PD‐1 checkpoint in cancer pathway and the Th17 cell differentiation pathway with the DMRs between AA and healthy controls (Figure 4E,F and Figure S3d). Further gene set enrichment analysis showed no significant difference in activation of the Th17 differentiation pathway (*p* = .19, Figure 4G) but significantly higher activation of the Janus kinase/signal transducer and activator of transcription (JAK/STAT) signalling pathway (*p* < .001, Figure 4H) in C01‐CAMK4‐TN cells from AA patients, compared with healthy controls. It was similar in the comparisons between C01‐CAMK4‐TN cells and other TN cells (Figure S3e,f). STAT3 phosphorylation in Jurkat cells treated with stattic (a small‐molecule inhibitor of STAT3 activation; Figure 4I). We found that stattic decreased the proportion of naïve T cells and was not obliterated by interleukin‐2 (IL‐2) (Figure 4I,J).

**FIGURE 4:**
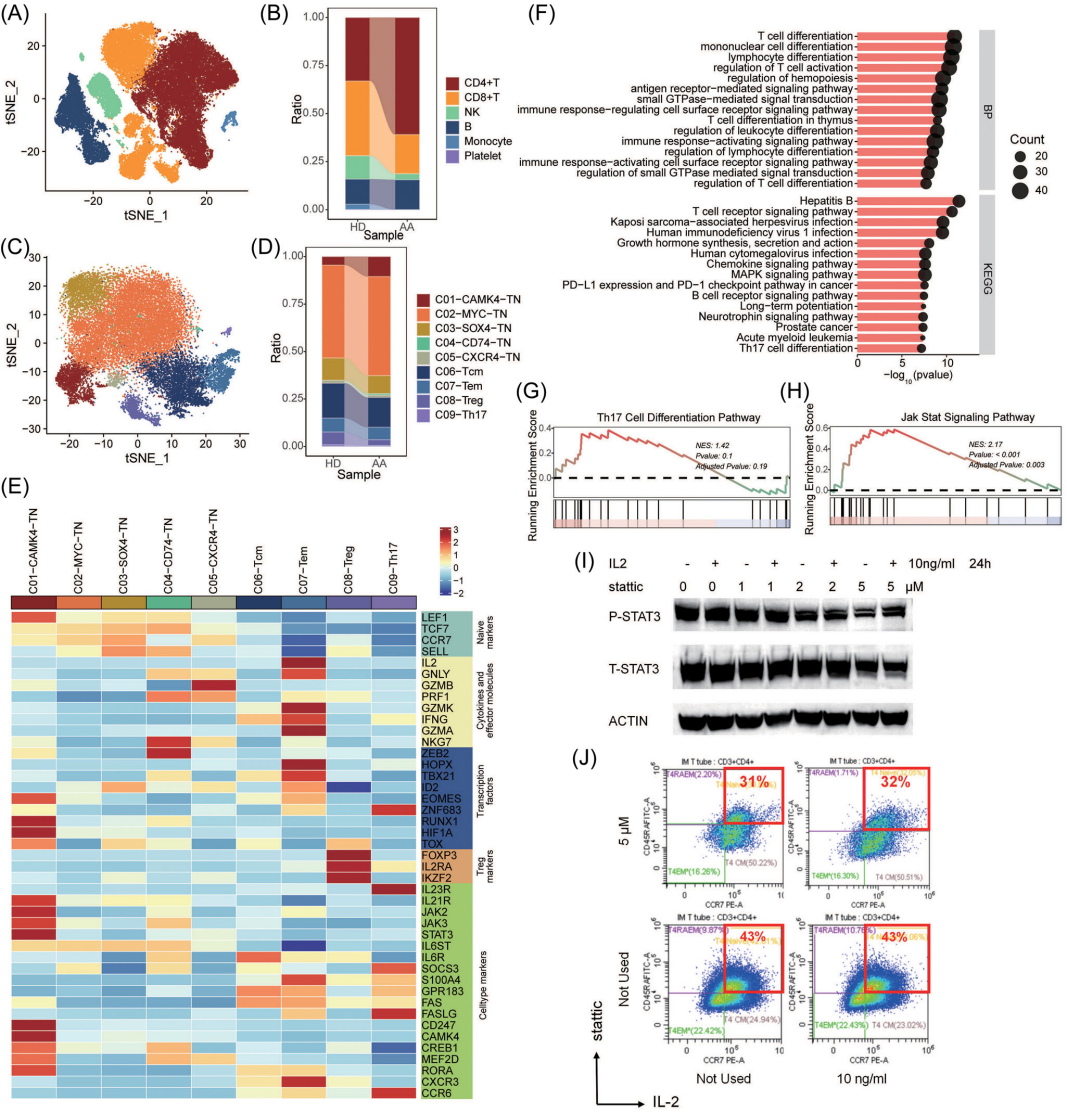
Aggregate analysis of single cells and cell culture. (A) t‐distributed stochastic neighbor embedding (tSNE) plot of 55 939 single cells distributed by annotated unsupervised clustering. (B) Alluvial plot of different proportion of cells between AA patients and healthy controls. (C) tSNE plot of the remaining 26 197 cells after subset CD4+ T cells. The annotation was conducted using well‐established markers. (D) Alluvial plot of different proportion of subdivided CD4+ T cells between AA patients and healthy controls. (E) Z‐score normalised mean expression of selected T‐cell function‐associated genes in each cell cluster. (F) ORA of differentially expressed genes in C01‐CAMK4‐TN cells between AA patients and healthy controls. (G) Gene set enrichment analysis (GSEA) of the Th17 differentiation pathway in C01‐CAMK4‐TN cells between AA patients and healthy controls. (H) GSEA of the JAK/STAT signalling pathway in C01‐CAMK4‐TN cells between AA patients and healthy controls. (I) Jurkat cells were treated with stattic and/or IL‐2. Cell extracts were analysed by immunoblotting with the indicated antibodies. (J) CD3+ cells in Jurkat cells treated with stattic and/or IL‐2 were measured by flow cytometry.

This study revealed the multi‐dimensional association of the T‐cell methylation‐transcriptome‐function phenotype in children with AA. Flow cytometry showed an aberrant balance of Th17/Treg cells in children with AA. DNA methylation variability is most enriched in Th17 cell differentiation, Th1 and Th2 cell differentiation, and PD‐L1 expression and PD‐1 checkpoint pathways. scRNA‐seq identified that the CAMK4 subtype in CD4+ naïve T cells was significantly higher in AA patients, compared to the healthy controls, accompanied by activation in the JAK/STAT signalling pathway. ORA of this specific subcluster further showed shared enrichment in the pathways with the DMRs. These findings suggest an association between T‐cell DNA methylation variability and pediatric AA, with a potential link to the JAK/STAT signalling pathway activation.

The JAK/STAT signalling pathway is at the core of T‐cell DNA methylation and plays pivotal but incompletely understood roles in determining CD4+ T cell fate as well as CD8+ T cell oligoclonal expansion.^5^, ^6^ T‐cell differentiation alterations are closely associated with the pathogenesis of AA^7^ as well as various autoimmune diseases.^8^ Our scRNA‐seq findings independently align with a prior study by Zhang et al., which reported CAMK4‐naïve T cells as a predisposition to proinflammatory pathogenesis with activation of the JAK/STAT signalling pathway in a separate pediatric AA cohort.^9^ CAMK4 is necessary during Th17 cell differentiation and drives autoimmune imbalance.^10^ Our findings, though preliminary and derived from a limited cohort, provided additional evidence to support autoimmune T cells as a key pathogenic driver in the progression of AA. We need to further study the specific modification changes of DNA methylation in the JAK/STAT signalling pathway.

## AUTHOR CONTRIBUTIONS

*Research design, acquisition, analysis and interpretation of data, drafting the article and revising it critically for important intellectual content*: Junchen Lai, Fangli Chen and Yan Miao. *Acquisition data and collecting specimen*s: Manpin Zhang. *Acquisition data*: Hua Zhu, Huanhuan Liang, Yingwen Zhang and Yanxin Li. *Collecting specimens*: Liting Yang and Dabin Tang. *Recruiting the patients and collecting specimens*: Chengjuan Luo, Changying Luo, Xiaodong Wang and Jing Chen. *Analysis and interpretation of data*: Yu Liu. *Critical revision and taking the responsibility for the integrity of the entire work as guarantor of the paper*: Xia Qin and Xinan Wang. All authors gave final approval of the version to be published.

## FUNDING INFORMATION

National Natural Science Foundation of China (81600094 and 81970114)

## CONFLICT OF INTERESTS STATEMENT

The authors declare no conflicts of interest.

## ETHICS STATEMENT

This study was approved by the institutional review board of Shanghai Children's Medical Centre (SCMCIRB‐K2017017) and conducted in accordance with the Declaration of Helsinki. Informed consent was obtained from the parents or guardians.

## Supporting information



Supporting Information

## Data Availability

The data that support the findings of this study are available on request from the corresponding author upon reasonable request.
