# Supplementary material for: DNA methylation variability in pediatric aplastic anaemia contributes to T‐cell differentiation
Source: Clin Transl Med. 2026 Jan 8;16(1):e70588. doi: 10.1002/ctm2.70588 (PMC12783688; doi:10.1002/ctm2.70588)
Supplement: Supplementary file 1 — Supporting Information [file CTM2-16-e70588-s001.docx]

*Overall study design*

We first performed flow cytometry in 76 pediatric AA patients and 20 healthy controls to detect the populations of T cells, B cells, dendritic cells (DCs), and natural killer (NK) cells and their subtypes. We compared the cell ratios and absolute counts between the patients and controls and also conducted an analysis to examine the potential correlation between these cells with disease severity and the treatments. Next, we examined DNA methylation status in CD3+ T cells purified from 5 patients and 2 controls by whole-genome bisulfite sequencing. We identified the pathways related to pediatric AA under biological processes by DNA methylation region enrichment analysis. To explore potential functional correlates of DNA methylation variability, we conducted single-cell RNA sequencing (scRNA-seq) in 2 AA patients and combined the data with 3 healthy pediatric samples from the GEO database (GSM6250006). We clustered T cells into gene expression subtypes and performed gene set enrichment analysis using the scRNA-seq data to identify dysregulated pathways. Integrated analysis of DNA methylation enrichment pathways and RNA dysregulated pathways suggested potential involvement of the JAK/STAT signaling pathway in T cells. Finally, we used a small-molecule inhibitor to test the functional role of this pathway in the Jurkat cell line.

*Cohorts*

We recruited 83 patients with acquired AA and 22 controls under 18 years old at Shanghai Children’s Medical Center, Shanghai Jiao Tong University School of Medicine, between January 2016 and October 2024. IBMFs and other types of bone marrow failures with susceptibility genes to hematopoietic malignancies, such as *GATA2* and *SAMD9/SAMD9L*, were excluded. Hepatitis-associated aplastic anemia (HAAA) was also excluded. The healthy controls were recruited via annual physical examinations. This study was approved by the institutional review board of Shanghai Children’s Medical Center and conducted in accordance with the Declaration of Helsinki. Informed consent was obtained from the parents or guardians.

*Flow cytometry*

Samples were incubated with the DuraClone antibody cocktail (Immune Monitoring, Beckman Coulter Life Sciences, Bangalore, India) to stain immune cells. T cells, B cells, DCs, and NK cells were identified using flow cytometry (11-Color DxFlex Flow Cytometer, Beckman Coulter, Shanghai, China) with fluorescent anti-human monoclonal antibodies against known cell surface markers (Beckman Coulter, Bangalore, India) listed in Supplementary Table 1. Single-color-stained VersaComp antibody capture beads (Beckman Coulter LifeSciences, Brea, CA/USA) were used for compensation. CytExpert software (version 2.0.0.283; Beckman Coulter, Brea, CA/USA) was used to analyze the results.

*T cell gating strategies*

As shown in Supplementary Figure 1a, CD3+ cells were defined as total T cells and divided into CD4+ T cells and CD8+ T cells using the CD4 and CD8 markers. Additionally, the CD4+ and CD8+ T cells were both divided into four subgroups as follows: naïve T cells (CD45RA+CCR7+) , central Memory T cells (CD45RA-CCR7+), effector memory T cells (CD45RA-CCR7-), and effector T cell subsets (CD45RA+CCR7-). CD4+Th cells were identified by excluding CD4+CD25highCD127low Treg cells. The CD4+ Th cells were further analyzed for CD183 and CD196 expression. Th1 like cells (CD4+CD25low/negCD127highCD183+ CD196-), Th2 like cells (CD4+CD25low/negCD127highCD183- CD196-), Th17 like cells (CD4+CD25low/negCD127highCD183- CD196+) could then be distinguished.

*Whole genome bisulfite sequencing (WGBS)*

The WGBS library was prepared using the EZ DNA Methylation Gold Kit (Zymo Research, Irvine, CA/USA) and sequenced using Illumina HiSeq 2500 (Sandor, Hyderabad, India). Raw reads were aligned with the hg19 (Homo sapiens) reference genome using the alignment tool Bismark (v0.14.3). The R package DSS v2.52.0 was used for DNA methylation analysis. All sites covered by at least 4 reads and FDR-adjusted q-values < 0.05 were used for downstream analysis. The minimum mapping quality was 20.

Differentially methylated regions (DMRs) were identified using the ‘callDMR’ function. Genomic coordinates were annotated using the R packages ChIPseeker v1.40.0 and org.Hs.eg.db v3.19.1. The ‘enrichGO’ and ‘enrichKEGG’ functions of the R package clusterProfiler v4.12.0 were used for enrichment analysis of gene ontology (GO) and Kyoto Encyclopedia of Genes and Genomes (KEGG) with default parameters.

*Single-cell RNA sequencing (scRNA-seq)*

Single-cell RNA sequencing was performed using the BD Rhapsody™ platform. Briefly, single-cell suspension was adjusted to appropriate volume for loading and capture using the BD Rhapsody™ Enhanced Cartridge Reagent Kit (Cat. No. 664887) and BD Rhapsody™ Cartridge Kit (Cat. No. 633733). Reverse transcription was performed using the BD Rhapsody™ cDNA kit (Cat. No. 633773). The BD Rhapsody™ WTA Amplification Kit (Cat. No. 633801) was used for DNA library construction. High-throughput sequencing was performed using the PE-150 mode.

*Pre-processing single-cell RNA-seq data*

The BD Rhapsody™ Sequence Analysis Pipeline 2.0 was used to process the raw data, demultiplex cellular barcodes, map reads to the hg19 reference genome, and generate a feature-barcode matrix. The output was converted into a Seurat object by the R package Seurat V4.4.0 for quality control. Cells that expressed fewer than 200 genes or with over 20% mitochondrial genes were excluded from the analysis. Single cells with over 3000 genes were also filtered out. Doublets were assessed using the DoubletFinder v2.0.4 R package, and few doublets (<10%) were observed outside of the single-cell population.

*Canonical correlation analysis (CCA), dimensionality reduction, and clustering*

Seurat was used to integrate samples and correct batch effects. The ‘NormalizeData’, ‘FindVariableFeatures’, ‘ScaleData’, and ‘RunPCA’ functions were used with default parameters. Clustering was conducted using the ‘FindClusters’ function and visualized using 2D t-Distributed Stochastic Neighbor Embedding. The ‘RunTSNE’ function was used to visualize and explore these datasets. The ‘FindAllMarkers’ function was used to identify marker genes of cell clusters. We annotated cell clusters based on the expression of known cell markers based on previous studies and cluster-specific genes.

*Functional enrichment analysis*

Using the ‘FindMarkers’ function in the R Seurat Packages, differentially expressed genes were identified between healthy controls and AA patients or between clusters. These identified genes were then used in the over-representation analysis. Functional enrichment was conducted and visualized using the R packages clusterProfiler v4.12.0 and ggplot2 v3.5.1.

*Cell culture and* *Western blot*

Jurkat cells (ATCC, VA/USA) were cultured in RPMI1640 medium (Gibco, CA/USA) supplemented with 1% penicillin/streptomycin (Beyotime Biotechnology, Shanghai, China) and 10% fetal bovine serum (Gibco, CA/USA). Antibodies against STAT3, phospho-STAT3, and β-tubulin were purchased from Cell Signaling Technology (Boston, MA/USA). Jurkat cells were treated with stattic (Targetmol, USA) for 24 hours, either in the presence or absence of recombinant human IL-2 (Proteintech, Wuhan, China).

*Statistical analysis*

The sample size in flow cytometry was not statistically predetermined but comprised all available cases, without further filtering. Comparisons between two groups were conducted using Student’s t-test for independent samples upon homogenous variance (as assessed by Levene’s test) and using Welch’s t-test for independent samples otherwise. One-way ANOVA was used for comparison involving multiple groups. A P value < 0.05 (2-sided) was considered statistically significant.



Supplementary Figure 1. Immune cells in control and patient cohorts. **a.** T cell gating strategies. **b.** Absolute counts of T cells. **c.** Percentages of B cells in lymphocytes. **d.** Absolute counts of B cells. **e.** CD4+/CD8+ ratio. **f.** Changes in CD4+ T cell proportion. **g.** Changes in CD8+ T cells.

CON, control; AA, aplastic anemia; NSAA, non-severe aplastic anemia; SAA, severe aplastic anemia; VSAA, very severe aplastic anemia; LYM, lymphocyte; Th17, T helper cells 17; Treg, T regulatory cells; IST, immunosuppressive therapy; TN, T naïve cells; TCM, T central memory cells; TEM, T effector memory cells; Teff, T effector memory cells.


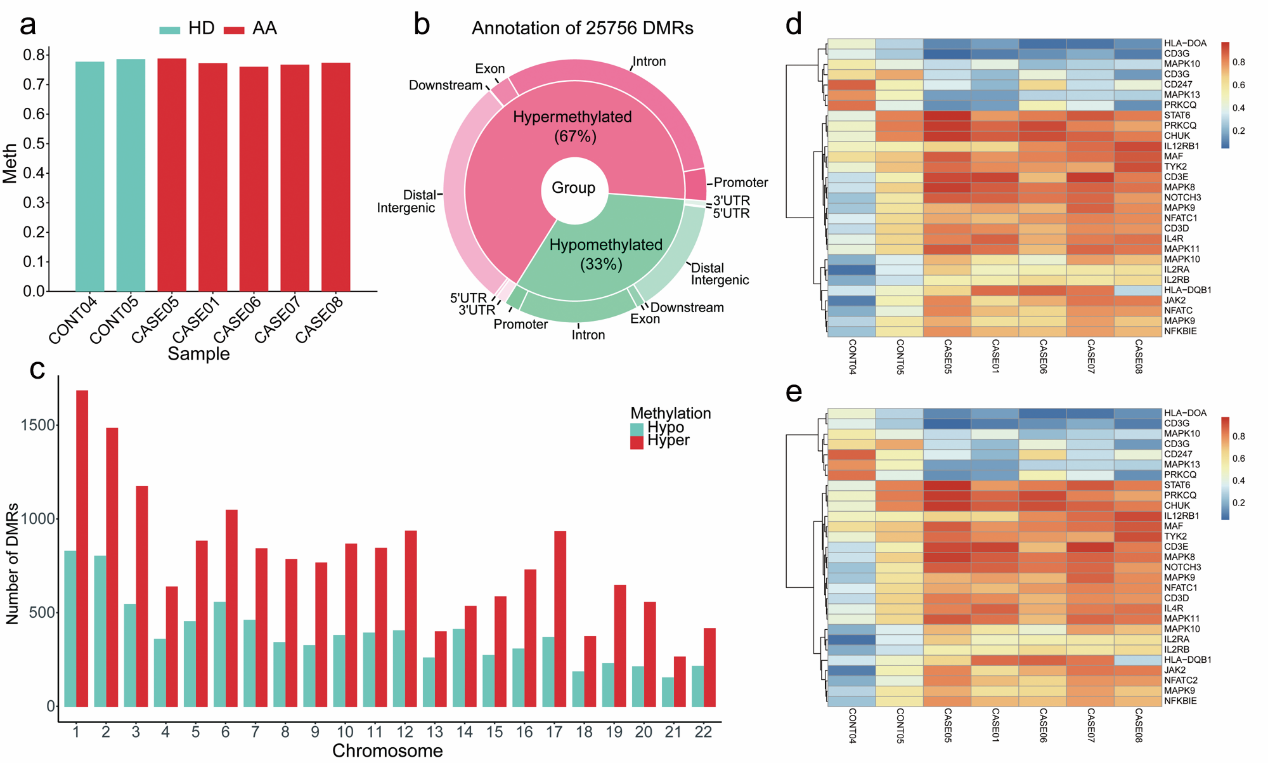
Supplementary Figure 2. Methylation difference between AA patients and healthy controls. **a**. Comparison of mean methylation among AA patients and healthy controls. **b**. Genomic distribution of hypermethylated and hypomethylated DMRs, in which DMRs in intron and distal intergenic were removed in following analysis. Hypermethylated: DMRs with higher degree of methylation in AA patients compared with healthy controls; Hypomethylated: DMRs with lower degree of methylation in AA patients compared with healthy controls. **c**. The distribution of DMRs on chromosomes. **d**. Hierarchical heatmap of methylation of genes from the th1 and th2 differentiation pathway. **e**. Hierarchical heatmap of methylation of genes from the PD-L1 expression and PD-1 checkpoint pathway in cancer pathway.


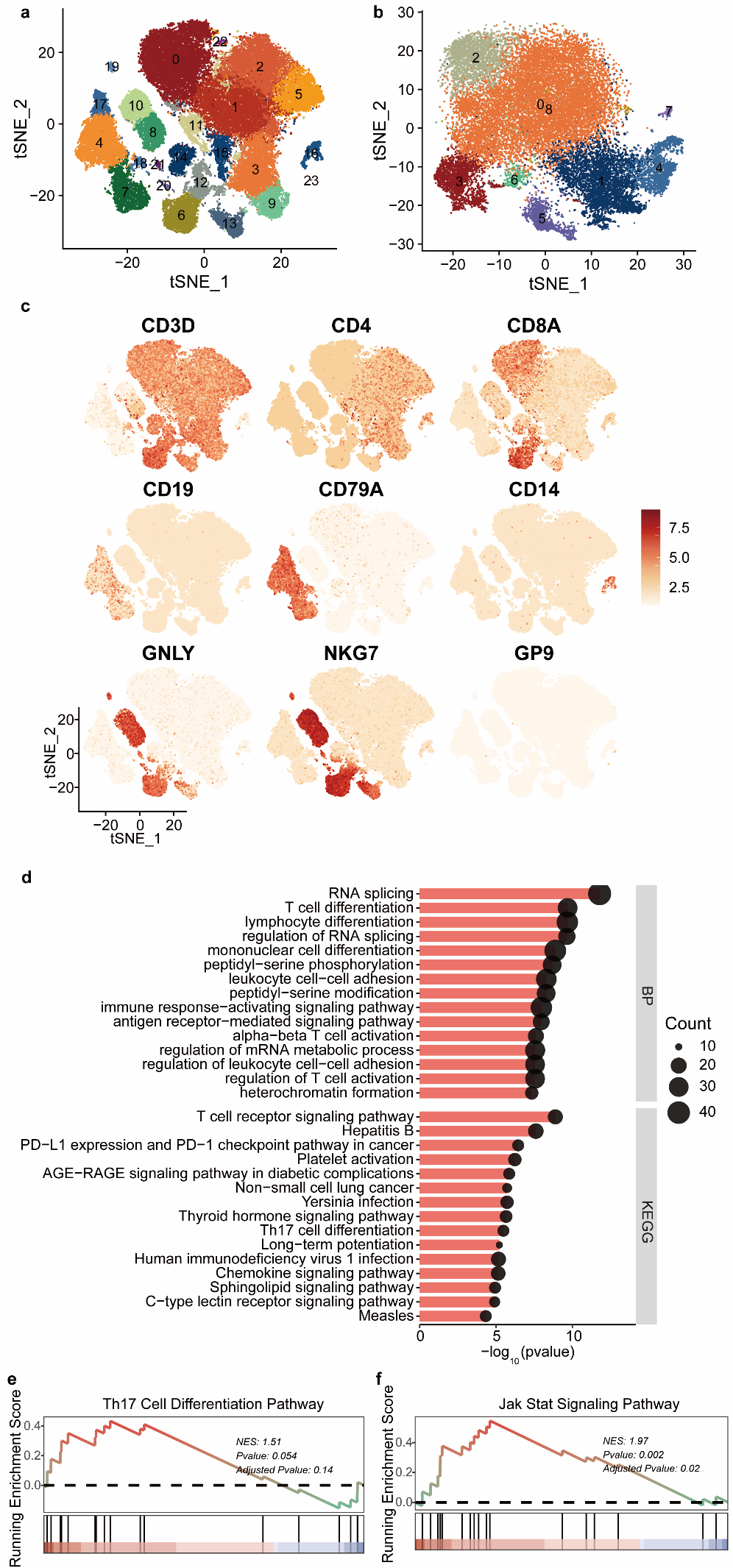


Supplementary Figure 3. Details of aggregate analysis of 55,939 single cells from two AA patients and three healthy controls. **a.** T-Distributed Stochastic Neighbor Embedding (tSNE) plot of 55,939 single cells showed 23 separate clusters. **b.** Differentially expression of representative markers of cells. **c.** TSNE plot of the remaining 26,197 CD4+ T cells showed 9 clusters. **d.** Over-representation analysis of differentially expressed genes in C01-CAMK4-TN cells between other naïve T cells. **e.** Gene set enrichment analysis (GSEA) of the Th17 differentiation pathway between C01-CAMK4-TN cells and other naïve T cells. **f.** GSEA of the JAK/STAT signaling pathway between C01-CAMK4-TN cells and other naïve T cells

Supplementary Table 1. Staining protocols of T/B/NK/DC/Monocytes cell subpopulation panel in flow cytometry

| Cellular subtype | Staining protocols | | | | | | | | | | |
| --- | --- | --- | --- | --- | --- | --- | --- | --- | --- | --- | --- |
|  | FITC | PE | ECD | PC5.5 | PC7 | APC | APC-A700/AF700 | APC-A750 | PB | KO | BV605 |
| IM Basic | CD16 | CD56 | CD19 | COUNT | CD14 | CD4 | CD8 | CD3 | HLA-DR | CD45 | CD38 |
| IM T | CD45RA | CCR7 | CD28 | PD1 | CD27 | CD4 | CD8 | CD3 | CD57 | CD45 |  |
| IM Th | CD183 | CD127 | CD45RA | CD25 | CD196 | CD4 |  | CD3 | HLA-DR | CD45 |  |
| IM B | IgD | CD21 | CD19 | CD5 | CD27 | CD24 |  | CD38 | IgM | CD45 |  |
| IM DC | CD16 | LIN |  | CD1c | CD11c | CD370 |  | CD123 | HLA-DR | CD45 |  |
| NK-kir | CD158b2 | CD158a | CD56 |  | CD314 | CD158i | CD159a | CD3 |  | CD45 | CD158e1 |

FITC, Fluorescein Isothiocyanate; PE, Phycoerythrin; ECD, Energy-Coupled Dye; PC, Phycoerythrin-Cyanine; APC, Allophycocyanin; AF, Alexa Fluor; PB, Pacific Blue; KO, Krome Orange; BV, Brilliant Violet.

Supplementary Table.2 Characteristics of patients anc controls

|  | Aplastic Anemia (N=76) | Controls (N=20) | P |
| --- | --- | --- | --- |
| Age, years  Average (range) | 8.3 (2 - 17) | 8.3 (5- 12) | 0.977 |
| Sex, n  Male / Female | 44 / 32 | 12/8 | 0.867 |
| Diagnosis, n (%)  VSAA  SAA  NSAA | 23 (30%)  20 (26%)  33 (43%) | / | / |
| Patients received CsA, n (%)  All  VSAA  SAA  NSAA | 56 (74%)  12 (52%)  18 (90%)  26 (79%) | / | / |
| Duration of CsA, median (range)  All  VSAA  SAA  NSAA | 2 (1 - 102)  1.5 (1-18)  2 (1-36)  3 (1-102) | / | / |

VSAA, very severe aplastic anemia; SAA, severe aplastic anemia; AA, aplastic anemia; CsA, Cyclosporin A.
